# Decolorization of the Teeth

**Published:** 1892-04

**Authors:** A. O. Rawls

**Affiliations:** Lexington, Ky.


### Decolorization of the Teeth.

BY A. O. RAWLS, D. D. S., LEXINGTON, KY.

Read before the Mississippi Valley Dental Association, March 11th, 1892.

By way of trying to add my mite to the general fund of experience given those present, I wish to say a very few words upon the subject of colorization, and dis or de-colorization of the dentine and enamel, wherein for the treatment of pulpless teeth, such antiseptics, stimulants, and germicides are used, the properties of which are known to produce a change of color in the tooth substance with which it comes in contact.

It is of common experience with the older practitioners and generally so with those of these latter days, that all preparations of Iodine soluble by contact or mixture with the fluids of tooth substance will change the color of those fluids. It is also known, or should be, that any preparation soluble or insoluble, in the nutrient fluids common to tooth substance will if pumped or even placed, in a fluid state in the crown cavity and root canal of a tooth, voided by hot air, Spunk, Bibulous paper, Alcohol or Ether, of a portion of its fluid contents, will most certainly presume to occupy the vacant space, and if of any marked difference in color from the tooth, will of necessity refract or reflect the same to observing eyes.

Terchloride of Iodine and Pyoktanin will each show their color in and throughout the tooth substance when used as above. Pyoktanin will show itself either blue or yellow, in accordance with which one of the preparations is used; no matter whether the tooth is anhydrous, only partly so, or deluged with saliva, mucus, or what not; it will fly apparently like the flash of strongest affinity, and we can hardly determine its bounds until we see its own definition. Such being the fact in so far as Pyoktanin is concerned, and partly, so far as the coloring properties of terchloride of iodine obtain, these articles would seem objectionable as remedial agents in the treatment of sepsis of tooth substance—objectionable only on account of their properties to maintain their color or change into that more objectionable.

Now let us see if we can remedy this. In trying I only give you my experience.

First. The pulp cavity and root canal being freed of its former occupants, and you opine from the character of the latter that the dentine needs throughout its open aspect germicidal treatment; then dry fairly well (not too dry nor too hot, for too much heat unbalances structure other than the germs,) the root canal; introduce upon the extreme point of a broach, touched in any of the volatile substances used in dentistry, a bit of, say Pyoktanin not larger than half a mustard seed, as near as possible to the apex of the root; stop the crown cavity, either loosely or tight, as the conditions require. In a few hours the discoloration will appear, this you wish to rid the tooth of, remove the stopping, fill the apex of the root with cotton and some resinous solution, and you can by the use of alcohol, a very dilute solution, in water of carbolic acid, or any of the hydrocarbons suitable for use, wash out with broach and cotton saturated with either all discolorization. If upon the other hand, you find that the dentine is fresh, clean and free from septic contact, and you wish only to treat an open wound at the end of the root, and wish to use in such treatment either one of the discoloring agents, and at the same time not discolor the tooth crown, then dry the crown cavity and root canal thoroughly, stop the end of the root temporarily, and while the tooth is dry and warm pump into the crown and root canal carbolated ethereal solution of Gum Copal or Sandarac, let this dry, remove the temporary stopping at end of root, and proceed with treatment for root membrane, periosteum and nerve tissue at end of root.

The very natural tendency of Pyoktanin is when stopped up in a tooth, to pass out when it meets moisture, therefore it would proceed in the direction of the cementum, and through it to the peridental membrane, and be lost in the circulation; but the animal fluids escaping from the cementum are slow to reach the crown of the tooth, and ere long become clogged up so that moisture cannot pass and carry back the substance of discoloration, hence it is better to wash it out before stopping the crown cavity. In some cases, pulpless teeth thus treated will if thoroughly freed from the stain of Pyoktanin, improve in color, becoming lighter and clearer. In choice of color I prefer the yellow preparation of E Mercks.

